# The Repair of Ruptured Crucial Ligaments by Open Operation

**Published:** 1902-12-13

**Authors:** 


### THE REPAIR OF RUPTURED CRUCIAL LIGAMENTS BY OPEN OPERATION.

At the last meeting of the Clinical Society three papers were read which, by a curious coincidence, enforced the same moral, namely the advantage of dealing by open operation with certain conditions which have often been treated, if one may so say, in the dark—by rest and massage in one case, by blind puncture in another, and by happy-go-lucky taxis in another.

Mr. Mayo Robson described the case of a miner, aged 41 years, who was admitted to the Leeds General Infirmary for lameness, the result of an accident nine months before. When admitted the symptoms showed that not only were all the ligaments of the knee relaxed but that the crucial ligaments had been ruptured. The joint was therefore opened by a semilunar incision carried across the front, dividing the ligamentum patellæ. The synovial membrane was found to be inflamed, and there was an excess of fluid in the joint. Both crucial ligaments were completely ruptured, having been torn from their upper attachments, and the torn ends were in a shreddy condition. They were stitched in position by means of catgut, the anterior ligament being stitched to the synovial membrane on the inner side of the external condyle, while the posterior, which was too short, was split in order to lengthen it, and was then fixed by sutures to the synovial membrane and cartilage on the outer side of the inner condyle. The wound was then closed. The plaster of Paris was kept on for a month, after which movement gradually returned under massage. When seen eleven months after the operation the patient was walking without a limp, could run, and could work eight hours a day at his old employment. The president, Mr. Howard Marsh, thought that as a rule it was not wise to open a joint when it was acutely inflamed, and said he had been much struck by the remarkable way in which ligaments sometimes became repaired, instancing the case of a boy whose knee-joint after injury was left in a flail-like state, yet six months later was found to be in a very serviceable condition. Mr. Robson concurred with this, but pointed out that in his case nine months after the accident the limb was still absolutely useless, and that the only alternative would have been amputation.

